# Dr. Henry Delos Blanchard

**Published:** 1912-08

**Authors:** 


Dr. Henry Delos Blanchard, Albany Medical College, 1882, of Portlandville, N. Y., died suddenly in a restaurant at Oneonta, June 26, from fatty degeneration of the heart. He was formerly president of the Otsego Co. Medical Society.

